# The Ketogenic Diet in Colorectal Cancer: A Means to an End

**DOI:** 10.3390/ijms24043683

**Published:** 2023-02-12

**Authors:** Magie Tamraz, Najib Al Ghossaini, Sally Temraz

**Affiliations:** 1 Department of Nutrition and Dietetics, American University of Beirut Medical Center, Riad El Solh, Beirut 1107, Lebanon; 2 Department of Internal Medicine, Ain Wazein Medical Village, Chouf 5841, Lebanon; 3 Department of Internal Medicine, American University of Beirut Medical Center, Riad El Solh, Beirut 1107, Lebanon

**Keywords:** colorectal cancer, ketogenic diet, low carbohydrate diet, ketones

## Abstract

Some diets, such as high lipid and high glucose diets, are known to increase the risk of colorectal cancer. On the other hand, little is known about diets that prevent colonic carcinogenesis. The ketogenic diet, which is characterized by high fat and very low carbohydrate content, is one such diet. The ketogenic diet decreases the amount of available glucose for tumors and shifts to the production of ketone bodies as an alternative energy source for healthy cells. Cancer cells are unable to use the ketone bodies for energy thus depriving them of the energy needed for progression and survival. Many studies reported the beneficial effects of the ketogenic diet in several types of cancers. Recently, the ketone body β-hydroxybutyrate has been found to possess anti-tumor potential in colorectal cancer. Despite its beneficial effects, the ketogenic diet also has some drawbacks, some of which are related to gastrointestinal disorders and weight loss. Thus, studies are being directed at this time towards finding alternatives to following a strict ketogenic diet and supplementing patients with the ketone bodies responsible for its beneficial effects in the hope of overcoming some potential setbacks. This article discusses the mechanism by which a ketogenic diet influences growth and proliferation of tumor cells, it sheds the light on the most recent trials regarding its use as an adjunctive measure to chemotherapy in patients with metastatic colorectal cancer, and it explains the limitations of its usage in metastatic patients and the promising role of exogenous ketone supplementation in this setting.

## 1. Introduction

Colorectal cancer (CRC) ranks third among cancers worldwide but is second in terms of mortality risk [1]. Despite advancement in surgery and chemotherapy, as well as implementation of targeted and immunotherapy, many CRC patients continue to have poor prognosis, and many seek alternative strategies or complementary treatments. One such alternative involves dietary intervention to enhance treatment and help control side effects of treatment. Additionally, risk of CRC has been positively correlated with specific dietary patterns [2] and elevated body mass index (BMI) levels [3]. For instance, a high fat diet-induced obesity also referred to as a Western diet, has been shown to augment the oncogenic potential of precursors of intestinal cells and to suppress immunity against tumor cells [4,5]. Moreover, elevated sugar diets leading to abnormal levels of fasting insulin and glucose have also been shown to increase risk of CRC [6]. The connection between carbohydrates and cancer has been described by Otto Warburg and Colleagues in 1923, who found a markedly increased consumption of glucose by tumors in comparison to the non-proliferating normal tissues [7,8,9,10]. Warburg observed that unlike normal cells that decrease glucose uptake and inhibit lactate production under aerobic conditions, tumor cells convert high amounts of glucose to lactate even in the presence of oxygen (aerobic glycolysis) [11,12]. It was found that aberrantly activated oncogenes and loss of tumor suppressors deregulates the import of glucose and amino acids into cancer cells are responsible for the increased uptake of glucose [9]. Furthermore, low muscle mass or sarcopenia is another important risk factor in CRC patients that is rarely diagnosed. Evidence has shown an association between sarcopenia and CRC incidence independent of obesity [13] as well as increased postoperative complications [14,15], and decreased disease-free and overall survival [16].

On the other hand, paucity of data exists on diets that could prevent or treat intestinal carcinogenesis. While abstinence and calorie deficit show potential in impairing glycolysis and inhibiting proliferation in CRC cells [17,18], implementation in cancer patients could pose many difficulties one of which is related to tumor cachexia. Cachexia is characterized by loss of skeletal muscle and weight that is attributed to decreased intake of food and an aberrant metabolism [19]. One nutritional intervention most likely to address this issue is the ketogenic diet (KD). KD has proven its success in the treatment of epilepsy [20]. A diet that is high in fat, normal protein and very low carbohydrate content has the potential to reverse the weight and muscle loss associated with cancer cachexia. Moreover, as the majority of CRC cells exhibit an altered metabolism that is expressed by increased glycolysis and decreased oxidative phosphorylation [21], the KD, which is distinguished by its very low carbohydrate content, decreases the amount of available glucose for tumors and shifts to the production of ketone bodies as an alternative energy source for healthy cells [22]. 

In this review, the process of ketogenesis is explained and the effect of KD as an alternative treatment choice in CRC patients is highlighted. The mechanism by which KD influences tumor growth and proliferation is thoroughly examined with emphasis on the future potentiality of this dietary intervention.

## 2. The Ketogenic Diet

**Figure 1:**
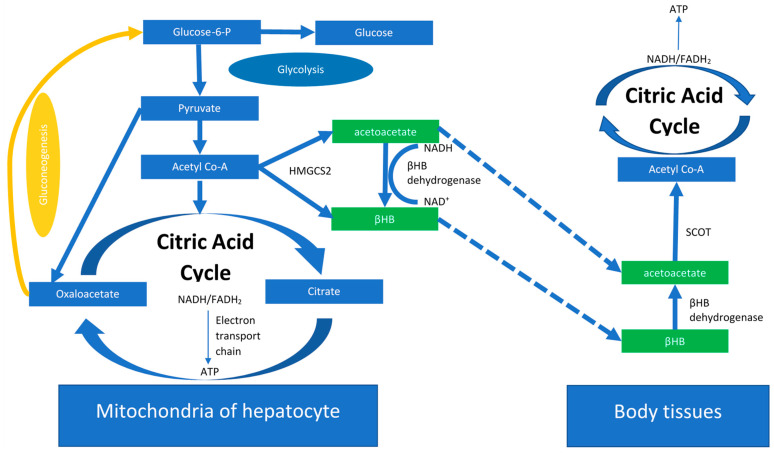
Ketogenesis is initiated upon deficiency in glucose intake. Ketone bodies are produced as a result which from mitochondria of hepatocytes which can later be used by other body tissues as a form of energy. ATP: adenosine triphosphate, βHB: β-hydroxybutyrate, HMGCS2: 3-hydroxy-3-methylglutaryl-CoA synthase 2, Glucose-6-P: Glucose-6-phosphate, NADH: nicotinamide adenine dinucleotide hydrogen, FADH_2_: D flavin adenine dinucleotide, SCOT: succinyl-CoA:3-ketoacid CoA transferase.

The ketogenic diet is distinguished by its very low carbohydrates (~5% of total caloric intake), moderate protein (~15% of total caloric intake) and high fat content (~80% of total caloric intake). The most common adaption of this diet involves a 4:1 ratio of fat to carbohydrate and protein [23]. When carbohydrate intake is insufficient, oxidation of fatty acids peaks and production of acetyl-CoA increases in the mitochondria of hepatocytes [24,25]. Acetyl-CoA then enters the citric acid cycle joined with oxaloacetate. When oxaloacetate is used up and its amount does not level up to the balance of the citric cycle, acetyl-CoA starts to make the ketone bodies acetoacetate and β-hydroxybutyrate (βHB) as an alternative source of energy for tissues outside the liver. These ketone bodies can control substrate use, inflammation, oxidative stress, catabolic processes, and gene expression [26]. This is accomplished with the help of the mitochondrial enzyme 3-hydroxy-3-methylglutaryl-CoA synthase 2 (HMGCS2) that activates the first step of ketogenesis [27]. βHB delivers more adenosine triphosphate (ATP) per mole of substrate as compared to pyruvate. Ketosis is achieved when blood βHB reaches concentrations ≥ 0.5 mmol/L [28]. The metabolic enzyme succinyl-CoA:3-ketoacid CoA transferase (SCOT) is not present in the liver, thus liver cells cannot use ketone bodies for energy and therefore acetoacetate and βHB can leave the liver and enter the bloodstream to be distributed to different body tissues including the brain with the help of monocarboxylate transporters [29,30]. βHB dehydrogenase can convert the ketone bodies to each other and this conversion reduces nicotinamide adenine dinucleotide (NAD+) to NAD+ (NADH). Ketones could then be used by brain neurons or other body tissues to reform acetyl-CoA which then enters the citric acid cycle again to generate ATP (Figure 1). However, tumors cannot make use of ketone bodies because they do not express one or more of the enzymes β-hydroxybutyrate dehydrogenase (β-OHBDH) or SCOT therefore depriving them from energy that they need for progression and survival [31].

### The KD Anti-Tumor Potential

The anti-tumor potential of KD was demonstrated in some in vitro studies. These studies revealed that tumor cell lines (glioma, breast, and colon) are not able to employ ketones as their dietary energy source when starved of glucose thus resulting in decreased glycolysis and tumor proliferation [32,33,34]. Moreover, a multitude of mouse studies supported the anti-tumor effects of KD in various types of cancers, including brain [35], prostate [36,37,38,39,40], breast [41], lung [42], gastric [43], and colon [44] cancers and its efficacy to inhibit tumor progression and prolong survival in mice with metastatic cancer [45]. 

Additionally, a few case reports and pre-clinical studies obtained promising results in cancer patients. The first attempt to treat cancer patients with a long-term controlled KD was reported by Nebeling et al. for two pediatric patients with astrocytoma [46]. Ketosis was maintained by consuming a 60% medium chain triglyceride oil-based diet. Results revealed a 21.8% average decrease in glucose uptake at the tumor site in both subjects. One of the patients exhibited significant clinical improvements in mood and new skill development during the study. She continued the KD for an additional 12 months, remaining free of disease progression [46]. In another case, a female patient with glioblastoma multiforme was treated with a KD which clearly demonstrated that this intervention is capable of stopping tumor growth [47]. However, the KD resulted in a 20% loss of weight over the treatment period of two months. In a pilot trial by Schmidt et al., 16 patients with advanced metastatic tumors and no conventional therapeutic options were instructed to follow a KD (less than 70 g carbohydrates per day) over a three-month period. The trial demonstrated the feasibility of KD with improvement in nearly all standard blood parameters and some measures of quality of life. Severe side effects were not observed except for constipation and fatigue [48]. Moreover, another safety and feasibility pilot trial of 10 patients with metastatic cancers demonstrated that an insulin-inhibiting diet is safe and feasible in selected patients with advanced cancer. The extent of ketosis, but not calorie deficit or weight loss, correlated with stable disease or partial remission. No unsafe adverse effects were associated with KD [49]. The ERGO trial examined the feasibility of an unrestricted ketogenic diet which provided 60 g of carbohydrates per day in 20 patients with recurrent glioblastoma [50]. The effects of a KD alone or in combination with bevacizumab was also explored in an orthotopic U87MG glioblastoma model in nude mice [50]. Median progression free survival (PFS) of all patients was 5 weeks and median survival from enrollment was 32 weeks. The trial allowed continuation of the diet beyond progression. Six of seven patients treated with bevacizumab and diet experienced an objective response, and median PFS on bevacizumab was 20.1 weeks. At 6 months, 43% of patients had no disease progression. In the mouse glioma model, KD alone had no effect on median survival, but increased that of bevacizumab-treated mice from 52 to 58 days (*p* < 0.05) [50]. No severe adverse events were associated with the diet. The results revealed that this diet is safe and feasible but probably has no significant effect as single agent in the treatment of recurrent glioma. 

## 3. Mechanism by Which KD Influences Growth and Proliferation of Tumor Cells

**Figure 2:**
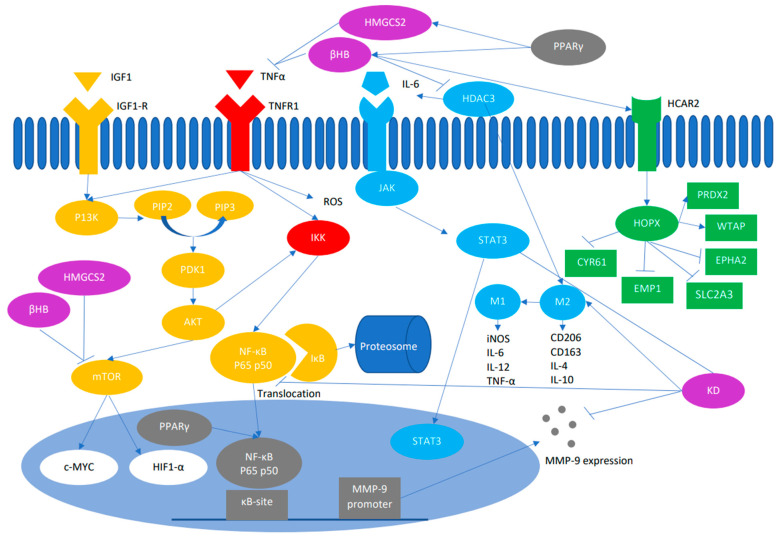
Depicts the major pathways implicated in CRC and the mechanism by which the KD or its products, the ketone body βHB and the enzyme HMGCS2, inhibit several genes and downstream effectors of these pathways. IGF1: insulin growth factor 1, IGF1-R: insulin growth factor receptor 1, TNFα: tumor necrosis factor alpha, TNFR1: tumor necrosis factor receptor 1, HMGCS2: 3-hydroxy-3-methylglutaryl-CoA synthase 2, βHB: β hydroxybutyrate, IL: interleukin, PPARγ: peroxisome proliferator-activated receptor gamma, HDAC3: histone deacetylase 3, HCAR2: hydroxycarboxylic acid receptor 2, JAK: Janus kinase, PI3K: phosphatidylinositol-3 kinase, PIP: putative plasma membrane intrinsic protein subtype, PDK1: 3-phophoinositide-dependent kinas 1, AKT: protein kinase B, mTOR: mammalian target of rapamycin, ROS: reactive oxygen species, IKK: IκB kinase, NF-κB: nuclear factor-κB, c-MYC: cellular myelocytomatosis oncogene product, HIF-α: hypoxia inducible factor alpha, MMP-9: matrix metalloproteinase 9, KD: ketogenic diet, TNFα: tumor necrosis factor alpha, CD163: Cluster of Differentiation 163, CD206: Mannose receptor C type 1, HOPX: Homeodomain-only protein, EPHA2: Ephrin type A receptor, EMP1: epithelial membrane protein 1, CYR61: cysteine-rich angiogenic inducer 61, SLC2A3: solute carrier family 2 member 4, PRDX2: peroxiredoxin 2, WTAP: Wilms tumor 1 associated protein.

One feature of cancer metabolism is an increase in glucose uptake, which leads to elevated insulin levels, which in turn elevates the insulin-like growth factor 1 (IGF1). Binding of insulin and IGF-1 to their receptors tyrosine kinases leads to autophosphorylation and subsequent activation of phosphoinositide 3-kinase (PI3K) [51]. PI3K in turn leads to activation and autophosphorylation of protein kinase B (AKT) pathway. Overactivation of PI3K/AKT pathway has been connected to low glucose settings and results in swift tumor cell death [52,53]. AKT stimulates mammalian target of rapamycin (mTOR). mTOR brings about aerobic glycolysis by through its influence on key glycolytic enzymes, specifically through its downstream effectors c-Myc and hypoxia inducible factor (HIF)-1a. c-Myc has main role in regulating metabolism specifically in response to changes in TME [54] while HIF-1a controls the intake of glucose and the expression of genes associated with glycolysis and energy metabolism [55]. On the other hand, the AKT/I*κ*B kinase pathway enhances angiogenesis and metastasis in CRC by stimulating nuclear factor-*κ*B (NF*κ*B) and β-catenin [56] (Figure 2). 

NF-κB is a heterodimer protein composed of two subunits, p65 and p50, which are needed for activating and translocating NF-κB to the nucleus [57]. Extracellular stimuli, such as a tumor necrosis factor receptor (TNFR), interacts with its ligand TNF to up-regulate the IκB kinase (IKK) complex [58]. IKK complex in turn phosphorylates p65/p50-bound IκB. Phosphorylated IκB is degraded via the ubiquitin-proteasome pathway consequently activating NF-κB. Activated NF-κB translocates to the nucleus where it activates down-stream genes expression that possibly enhances inflammation and the initiation and progression of cancer (Figure 2). Activation of this pathway results in increased expression of cyclin D1, cyclin E, and cyclin-dependent kinase (CDK)-2, as well as IL-6 and Myc. IL-6 activates signal transducers and activators of transduction-3 (STAT3). IL-6/STAT3 signaling has an important consequence on tumor-infiltrating immune cells in the tumor immune microenvironment in CRC [59]. 

Histone deacetylase 3 (HDAC3) controls the secretion of IL-6 and takes part in the enhancement of the protumor M2 phenotype of macrophage [60]. Tumor-associated macrophages (TAMs) are polarized into the M1 which are classically activated and inhibit tumor development or the M2 which are alternatively activated and enhance tumor progression and metastasis [61]. The M1 phenotype macrophage up-regulated the expression of inducible nitric oxide synthase (iNOS), IL-12, MHC, and CD16/32 molecules and produced proinflammatory factors, such as IL-6, IL-12, and TNF-α, which can initiate immune response [62]. M2 phenotype on the other hand produced Mannose receptor C type 1 (CD206), Cluseter of differentiations 163(CD163), arginae-1, IL4, and IL10, which are involved in the anti-inflammatory response and wound curing [63]. NF-κB is also involved in the expression of matrix metalloproteinase (MMP)-9 (Figure 2). MMP9 is a proteinase that can digest the extracellular matrix and basement membranes and type IV collagen underlying the blood vessels which eases the migration of aggressive cancers [64]. 

In vivo, the KD down-regulated the protein expression of MMP-9 and enhanced the M2 to M1 TAM polarization. Moreover, the levels of HDAC3/PKM2/NF-κB 65/p-Stat3 proteins decreased in the KD group [65]. In differentiated intestinal cells, enrichment of βHB and expression of HMGCS2 were elevated. Treatment with the ketone body βHB increased intestinal cell differentiation but inhibition of the enzyme HMGCS2 decreased intestinal cell differentiation. Treatment with βHB or overexpression of HMGCS2 induced CDX2 expression and inhibited mTOR signaling [66]. Additionally, it was found that HMGCS2 overexpression decreased TNFα-induced apoptosis and expression of pro-inflammatory chemokines (CXCL1-3). Treatment with βHB decreased TNFα-induced apoptosis in the intestinal epithelial cells and decreased the production of reactive oxygen species (ROS) that are generated by TNFα [67]. Recently, it was demonstrated that βHB induced up-regulation of the (homeodomain-only protein) *HOPX* gene. βHB acts through the surface receptor (hydroxycarboxylic acid receptor 2) HCARr2 to influence *HOPX* expression and decrease epithelial growth [68]. Epigenetic silencing of *HOPX* promotes progression in CRC [69]. A similar phenotype was observed in human CRC models expressing both HCAR2 and *HOPX*, with serum from 41 patients with CRC further indicating that serum levels of βHB correlate with *HOPX* expression in these patients [68]. Multiple combination treatment based on KD in this work also demonstrated additive effects in tumor suppression with different mechanisms, such as the combination with glucose restriction, histone deacetylase inhibitors (vorinostat and butyrate) and DNA methylation inhibitor (5-azacitidine) [70].

## 4. KD as Adjunct Treatment in CRC Patients

KD diminished tumor growth and prolonged survival in mouse models. In mouse xenograft models of colon cancer cell lines, a KD composed of omega3 and medium chain triglycerides and another composed of lard significantly delayed the tumor growth compared to standard diet group [71]. In another model, mice that were fed a ketogenic formula were found to preserve their body, muscle, and carcass weights. Moreover, tumor weight and plasma IL-6 levels were significantly lower in this group [72]. Mice fed a KD had elevated ketone body levels and slight decrease in serum insulin and significantly larger necrotic areas and less vessel density compared to the standard diet group [73]. Similarly, another in vivo study on antitumor efficacy of KD on transplanted CT26+ tumor cells in BALB/c mice revealed that the KD group had significantly higher blood β-HB and lower blood glucose levels compared to the standard group [65]. In vitro, βHB reprogrammed energy metabolism and repressed CRC proliferation [74].

Human studies on the prognostic effect of low carbohydrate diets in patients with CRC remains low. Stage III CRC patients consuming a diet high in carbohydrates and glycemic load had decreased survival [75]. The effect of a diet low in carbohydrates and macronutrients revealed that intake of carbohydrates was related to higher CRC mortality in stages I to III [76]. Results of a population-based case-control study of CRC showed decreased risk of cancer deaths in those who consumed the KD [77]. One possible explanation behind these results could be attributed to the fact that diets high in carbohydrates elevate blood glucose levels that induce insulin production. In turn, insulin has the potential to encourage cell proliferation, to impede apoptosis, and to increase carcinogenesis through insulin growth factor 1 (IGF1) by diminishing the amount of IGF binding proteins [78]. Additionally, fat, specifically plant fats such as oils and nuts, has the potential to decrease level of insulin circulating in the blood as well indicators of inflammation [79]. Thus, a KD which is rich in fat specifically from plant origin and low in carbohydrates has insulin sensitizing capability which could affect CRC progression [80,81]. 

In addition to the disease itself, the side effects of chemotherapy and radiotherapy can be very incapacitating and severely influence quality of life (QoL). Some of these side effects include nausea, vomiting, fatigue, hair loss, nerve and muscle damage, cognitive impairment and weight loss [82]. Thus, any intervention that has the potential to ease these side effects is valuable. A low carb diet and a KD in specific improved the self-reported QoL among CRC patients and had the potential to normalize body weight [83]. Additionally, KDs control the side effects of chemotherapy, specifically nausea and fatigue, and prevent the loss of lean muscle [84,85]. Another prospective trial studied the clinical response of stage IV CRC on chemotherapy to a KD administered for a period of 1 year receiving chemotherapy [86]. Compared to patients on chemotherapy alone, the KD group showed a significantly higher overall response rate of 60% compared to 21% in the control group.

Cachexia and unintentional weight loss are grave complications that can negatively influence the prognosis of CRC patients. The KETOCOMP study which is a prospective trial that studied the effects of a KD on body composition in rectal patients receiving curative radiotherapy revealed that a KD significantly reduced body weight and fat mass averaging 0.5 and 0.65 kg/week [87]. There was a speedy loss of intracellular water resulting from depletion of muscle glycogen and water; however, skeletal muscle tissue was preserved. Pathologic tumor responses were higher in patients following the KD. The study also revealed a significant association between weight loss and severity of overweight in such a way that the majority of weight lost was seen in those who were overweight or obese prior to diet initiation. KD’s ability to counteract cancer cachexia might be related to βHB’s anti-inflammatory effect. IL-6 along with other inflammatory cytokines encourage muscle breakdown by triggering the ubiquitin-proteasome pathway. βHB is able to inhibit the production of interleukin (IL)-1β and IL-18 through NOD-, LRR- and pyrin domain-containing protein 3 (NLRP3) inflammasomes in human monocytes [88] and to decrease mRNA expression of the inflammatory cytokine IL-6 [72] therefore suggesting that βHB induction by consumption of KD may overwhelm the systemic inflammatory response and hinder cancer development. 

Recently, it was reported that ketogenesis regulates the tumor microenvironment (TME) in CRC and has potential to overcome immunosuppression and enhance survival. This study revealed that the products of ketogenesis, the ketogenic enzyme HMGCS2 or the ketone body βHB decreased expression of Kruppel Like Factor 5 (KLF5), which binds the C-X-C Motif Chemokine Ligand 12 (CXCL12) promoter and induces CXCL12 expression in cancer-associated fibroblasts. Low expression of KLF5 improves the immunosuppressive TME, augmented penetration of natural killer and cytotoxic T cells, and improved the antitumor effects of Programmed cell death protein 1 (PD-1) blockade in murine-derived colorectal cancer. This reveals that down-regulation of de novo ketogenesis in the TME is a vital step in CRC progression [89].

So far, and especially with regards to CRC, it should be pointed that diet quality is important as KDs with large quantities of artificial foods may have a negative influence on the gut microbiome and offset the otherwise beneficial effects of ketosis [90]. This was demonstrated in a recent study that found low carbohydrate diets that are enriched with animal products are associated with increased CRC risk and these adverse associations could be attenuated by plant fat consumption as a substitute for animal products [91]. Another study also showed that a diet rich in plants sources of fat and protein and low in carbohydrates was correlated with decreased CRC-specific death [76].

## 5. The Downfalls to the Clinical Application of KD

To achieve therapeutic ketosis, strict dietary compliance is required and this is often difficult specifically in terminally ill patients such as metastatic CRC patients. One review reported that the studies implementing the KD had low adherence to KD but the drop-out rates varied greatly between the different studies. The major reason behind low adherence were related to limitations in monitoring and delivery, unpalatability of meals and difficulty integrating the dietary pattern into family life [92]. Weight loss is another major drawback of KD since malnourishment, sarcopenia and cancer cachexia diminish quality of life and affect clinical outcome. Three randomized controlled studies in breast, prostate and ovarian/endometrial cancer reported significant weight loss in patients following the KD compared to the control group [93,94,95]. Weight loss was reported to be 12.1 kg compared to 0.5 kg in the control group during a 6 month period [93], whereas it ranged between 6.1 and 6.3 kg in the intervention group receiving the KD for a period of 3 months compared to 1.3–3 kg in the control group [94,95]. Additionally, a drawback of a diet that is low in carbohydrates involves the possible loss of skeletal muscle protein as a means to compensate for the amino acids needed to produce glucose. Specifically, the nervous system cannot use fat for energy and therefore has a mandatory need for glucose which it will obtain from amino acids. However, the body adapts to the low carbohydrate diet by reducing the brain’s requirement for glucose thus limiting the need for protein to provide the amino acids needed for glucose synthesis. Soon, the liver starts synthesizing ketones which could replace up to about 70% of the brain’s need for glucose, thereby decreasing skeletal muscle breakdown [96]. As depicted by one randomized controlled trial and several controlled trials, the changes in body composition after implementing the KD were associated with fat loss. Cohen et al. revealed that the intervention group lost fat at a rate of 5.2 kg compared to 2.9 kg in the control group, while the lean body mass was conserved [95]. Similar results were achieved by Ok et al. which reported fat losses of 1.9 kg in the intervention group but no loss of lean mass [97]. Klement et al. also reported that rectal cancer patients had a significantly greater loss of fat mass occurring in the KD group, without significant differences in lean mass [98]. Another potential drawback of a KD is the decreased intake of non-digestible carbohydrate which may elevate the risk of lower gastrointestinal tract disorders particularly constipation [99,100,101]. Moreover, low intake of fruit and vegetables rich in potassium could result in unwarranted bone loss due to the release of calcium-containing alkalizing salts to neutralize the acidity resulting from the high intake of protein associated with a KD. This could place patients at an increased risk of osteoporosis [102]. However, addition of foods rich in potassium can decrease the use of bone material to produce calcium in order to neutralize the extra acid.

Based on this, the use of exogenous ketone supplementation such as ketone esters (liquid form), ketone salts (powder form), and medium chain triglycerides (MCTs) which induce ketosis in a similar way that a KD might is justified. The making of β-HB from exogenous ketone supplementation is not influenced by carbohydrate intake, and thus administration of these supplements would reach therapeutic ketosis by following a normal healthy diet that does not involve carbohydrate restriction. MCTs are quickly absorbed, energy-dense, dissolve in water and have no taste. Supplementation with exogenous MCTs as a source of dietary fat was not well tolerated as high consumption of MCTs results in gastrointestinal side effects including diarrhea, dyspepsia, and flatulence. Oral administration of ketone esters fully metabolizes to βHB and acetoacetate. The two most prominent forms of ketone esters include (R)-3-hydroxybutyl (R)-3-hydroxybutyrate ketone monoester (KME) and R,S-1,3-butanediol acetoacetate ketone diester (KDE). Ketone esters are safe, tolerable, and effective ketogenic mediators [103,104,105]. An adequate dose of ketone salts or in combination with other exogenous ketone supplements, such as ketone esters and MCT may pose as a safe and successful way to reach ketosis [103,106].

Beside the greater feasibility of using exogenous ketone supplementation than following a strict KD diet [107], several differences exist between the two methods. While endogenous KD elevates blood β-HB levels to 0.5–3.0 mmol, exogenous supplementation elevates blood β-HB levels to 0.3–1.0 mmol [108]. To reach ketosis by following KD, a couple of days are needed whereas with exogenous ketone supplementation β-HB levels are elevated acutely. Additionally, glucose concentrations in the blood are different as a result of the diverse carbohydrates requirements. However, implementing either the KD or using exogenous supplementation contribute similar anabolic and catabolic effects [109]. 

## 6. Conclusions

Colorectal cancer is one type of cancer which is influenced by dietary patterns and body mass index. The majority of CRC cells exhibit an altered metabolism that is expressed by increased glycolysis and decreased oxidative phosphorylation. The KD, which is characterized by very low carbohydrate and high fat intake, has the potential to provide energy in the form of ketone bodies to all normal cells and deprive CRC cells of glucose which is needed for proliferation and metastasis. Moreover, the KD and specifically the ketone body βHB were found to play major role in several pathways such as IGF, PI3K, mTOR, and TNFα. Recently, the ketone body βHB exhibited the ability to suppress epithelial cell proliferation and inhibit tumor growth. The ketone body acts on cancer cells through regulation of Hopx, a known regulator of CRC. Furthermore, βHB requires a surface receptor Hcar to induce Hopx expression and suppress proliferation of intestinal epithelial cells [110]. 

Current evidence on the role of KD in CRC comes mainly from in vivo or in vitro studies and few human trials. In these studies, KD improved quality of life and attenuated many of the side effects of cancer treatment. Although there is still limited evidence to support the use of KD as an adjunct therapy in mCRC patients, the recent discoveries of the potential role of KD specifically βHB in CRC, are encouraging and many trials investigating the efficacy of this dietary therapy are underway. Several registered clinical trials are currently ongoing to investigate the case for a KD as a supportive therapeutic option in oncology mainly in breast, brain, prostate, and renal cancers. It is necessary that following the finding of the important role of βHB in CRC that future trials study its effect in complementation with standard treatment for CRC. 

Implementing the KD is not without disadvantages. Problems with palatability and strict dietary restrictions increase the dropout rate. Additionally, some reported side effects including gastrointestinal side effects such as constipation and weight loss prevent patients from adhering to the diet. To circumvent the downfalls of KD, exogenous ketone supplementation, providing adequate amounts of the ketone body βHB, is starting to be put into use. It is likely that future implementation of KD will be in the form of exogenous ketones where they will be administered alongside standard therapy in mCRC as they might pose as a more viable alternative to the KD. Additionally, another advantage of ketone supplementation is their tolerability and their ability to be formulated and titrated in a way that minimizes their gastrointestinal side effects, all of which pave the way for an acceptable and tolerable option for mCRC patients. Recently, a beneficial association between KD and epigenetic modulation has surfaced. Implementation of KD resulted in changes in DNA methylation, histone modifications and miRNA levels [111]. Data on this aspect are still at their very early stages and future studies may be directed into investigating this field further. 

## Data Availability

Not applicable.
